# Effect of Ketorolac on Pharmacokinetics and Pharmacodynamics of 5-Fluorouracil: In Vivo and In Vitro Study

**DOI:** 10.1155/2022/5267861

**Published:** 2022-09-21

**Authors:** Motahare Atefinezhad, Ahmadreza Moghadamnia, Seyed Parsa Eftekhar, Ali Akbar Moghadamnia, Sohrab Kazemi

**Affiliations:** ^1^Department of Pharmacology, Ayatollah Amoli Branch, Islamic Azad University, Amol, Iran; ^2^Student Research Committee, Health Research Institute, Babol University of Medical Sciences, Babol, Iran; ^3^Department of Pharmacology and Toxicology, School of Medicine, Babol University of Medical Sciences, Babol, Iran; ^4^Cellular and Molecular Biology Research Center, Health Research Institute, Babol University of Medical Sciences, Babol, Iran

## Abstract

**Background:**

This study aimed to evaluate the impact of ketorolac on the pharmacokinetics of 5-FU and its effect on the efficacy of 5-fluorouracil (5-FU) on the HT-29 cell line.

**Methods:**

Cell culture: the HT-29 cell line was treated with different concentrations of 5-FU, ketorolac, and combination of 5-FU and ketorolac for 24 and 48 hours. The cell viability (%) was calculated by the MTT assay. Animal study: rats were randomly divided into control and pretreatment groups. The control group received physiological saline, whereas the pretreatment group received ketorolac by intraperitoneal (i.p.) injections on a daily basis for 14 days. On the 15th day, both groups received 5-FU (i.p.). Blood samples were collected at different times for HPLC analysis, and 5-FU pharmacokinetic parameters were calculated.

**Results:**

At cell culture study, in a certain concentration range, combination therapy showed synergistic effects (<0.05). However, at concentrations above this range, combination therapy showed antagonistic effects on 5-FU efficacy (<0.05). According to the pharmacokinetic analysis, pretreatment with ketorolac resulted in a significant increase in AUC, *C*_max_, and *T*_max_ of 5-FU (<0.05) and a significant decrease in V/F and Cl/F of 5-FU (<0.05).

**Conclusions:**

Combination therapy with ketorolac and 5-FU, depending on time and concentration, has a synergistic effect on reducing the viability of cancer cells. Also, ketorolac is able to alter the pharmacokinetics of 5-FU. Since there is a close relationship between pharmacokinetic parameters of 5-FU and its effectiveness/toxicity, it seems that these changes are towards creating a synergistic effect on 5-FU cytotoxicity. These results suggest the need to optimize the dose of these drugs in order to increase clinical efficacy and reduce the toxicity associated with them.

## 1. Introduction

Despite the availability of new anticancer drugs, 5-fluorouracil (5-FU) as an old and well-known agent in cancer chemotherapy is still used in colorectal, breast, stomach, and pancreatic cancers. 5-FU-based chemotherapy needs further investigation in two aspects. First, the response rate to monotherapy with 5-FU is limited for several reasons, including pharmacokinetic properties (rapid metabolism and short half-life) and the rapid development of acquired resistance. Moreover, further increase of its dose leads to inevitable side effects [1–4]. Second, about 3 to 5% of patients have low activity of dihydropyrimidine dehydrogenase (the rate-limiting enzyme in the biotransformation of 5-FU), the reason for which can be traced to the polymorphism phenomenon. In other words, polymorphisms in dihydropyrimidine dehydrogenase (DPD) may result in a decrease or loss of DPD enzymatic activity and consequently decrease of 5-FU metabolism. This process leads to the accumulation of 5-FU which can cause significant toxicity, including leukopenia and thrombocytopenia [5].

Therefore, to prevent treatment failure and adverse effects, it is necessary to take appropriate considerations. In recent years, the role of nonsteroidal anti-inflammatory drugs (NSAIDs) in cancer management has been considered due to various mechanisms such as anti-inflammatory effects, inhibition of angiogenesis, induction of apoptosis and sensitivity to anticancer drugs, and enhancement of cellular immune responses. Acute inflammation has been shown to be one of the body's natural defense mechanisms, and the body recovers spontaneously after a short-term inflammatory response. However, long-term chronic inflammation may lead to carcinogenesis or worsening the prognosis of cancer by inducing cell proliferation, angiogenesis, and metastasis, as well as reducing the response to the immune system and chemotherapeutic agents. In fact, many molecular targets and signaling pathways in cell proliferation, angiogenesis, and apoptosis are common to inflammation and carcinogenesis, and chronic inflammation plays a role in malignant changes by disrupting the regulation of these targets and pathways [6–8]. Furthermore, due to the known effectiveness of NSAIDs in controlling pain, it seems that these drugs can counteract the undesirable effect of pain on the prognosis of malignancies as much as possible by overcoming the increase in endogenous opioid levels during the pain process and consequently, prevention of the activation of peripheral *µ*-opioid receptors and signaling pathways that affect tumor progression [9].

In addition to the role of NSAIDs in cancer management, these drugs are able to alter the pharmacokinetics and pharmacodynamics of other drugs, such as some chemotherapeutic agents [10]. Studies have evaluated the use of NSAIDs in combination with cancer chemotherapy in vitro and in vivo, and combination therapy has shown promising results in terms of effectiveness. It seems that combination of 5-FU with NSAIDs can provide a better prognosis in treatment of cancer. However, possible side effects cannot be ignored.

Ketorolac is a nonselective nonsteroidal anti-inflammatory drug with potent analgesic effects (closely to opioids) and moderate anti-inflammatory activity and plays an important role in pain alleviation in patients with advanced cancer. It is an excellent alternative to oral NSAIDs and makes possible to reduce the dose and adverse effects associated with opioid analgesics [11, 12]. Therefore, it may be coadministered with chemotherapeutic agents (concurrently or sequentially) to manage cancer-related pain. Accordingly, the interaction between ketorolac and chemotherapy drugs needs further investigation. The present study was carried out to investigate the effect of ketorolac on the pharmacokinetics of 5-FU in rats and also the effect of ketorolac on the efficacy of 5-FU on human colorectal adenocarcinoma cell line HT-29 [13].

## 2. Materials and Methods

### 2.1. Cell culture Study

#### 2.1.1. Cell Line and Chemicals

The human colorectal adenocarcinoma cell line HT-29 was obtained from the Pasteur Institute Cell Bank (Iran, Tehran) and was maintained in accordance with the instructions provided by the American Culture Collection. Culture medium RPMI 1640, fetal bovine serum (FBS), and penicillin-streptomycin (pen-strep) were purchased from BIO-IDEA (Iran). Thiazolyl blue tetrazolium was provided from Life Biolab (Germany). Trypsin and DMSO were purchased from Merck (Germany). 5-FU and ketorolac were obtained from Korea United Pharm (South Korea) and Caspian Tamin (Iran), respectively.

#### 2.1.2. Cell Culture

HT-29 cell line was cultured in RPMI 1640 medium supplemented with 10% (v/v) FBS and 1 (v/v) penicillin-streptomycin, under standard conditions (saturated humidity atmosphere containing 95% air and 5% CO_2_ at 37°C). At 75% of the junction, cells were harvested using 0.25% trypsin and were planted on a 96-well plate. The cells were allowed to attach to the surface for 24 hours before exposure to the drugs.

#### 2.1.3. Treatment and MTT Assays

The cells were treated with different concentrations of ketorolac, 5-FU (as positive control), and combination of ketorolac and 5-FU for 24 and 48 hrs. The concentrations of ketorolac were 0.005, 0.01, 0.02, 0.04, and 0.08 millimolar (mM). The concentrations of 5-FU were 0.007, 0.0137, 0.027, 0.055, and 0.11 mM. The concentrations of combination regimen of ketorolac and 5-FU were 0.0025 mM ketorolac +0.0035 mM 5-FU, 0.005 mM ketorolac +0.007 mM 5-FU, 0.01 mM ketorolac +0.0137 mM 5-FU, 0.02 mM ketorolac +0.027 mM 5-FU, and 0.04 mM ketorolac +0.055 mM 5-FU. Nontreated cells were considered as negative control. The MTT colorimetric assay was used to evaluate the effects of ketorolac, 5-FU, and combination therapy on the HT-29 cell line. For this purpose, after the cell treatment, the contents of 96-well plate were carefully removed and MTT (thiazolyl blue tetrazolium bromide) dye was added to it. Then, it was incubated for 4 hours under standard conditions. In the next step, the MTT dye was separated and the formazan crystals produced by the living cells were dissolved in DMSO. Finally, the absorbance of the samples was determined using an ELISA reader at 570 nm. The above steps were repeated at least 3 times for different concentrations of each group. Cell viability (%) and also IC-50 levels of ketorolac and 5-FU were calculated. The data were analyzed by one-way ANOVA using Prism 8.0 software, and the significance level was set at*p*-value ≤0.05. Cell viability (%) = (mean absorbance in the test group/mean absorbance in the negative control group) × 100.

### 2.2. Animal study

#### 2.2.1. Chemicals

5-Chlorouracil (5-ClU), ammonium acetate, DMSO, HPLC grade methanol, HPLC grade acetonitrile, and HPLC grade water were purchased from Merck (Germany). 5-FU and ketorolac were obtained from Korea United Pharm (South Korea) and Caspian Tamin (Iran), respectively.

#### 2.2.2. Animals

This study was carried out on 12 adult male Wistar rats with an average weight of 200 ± 20 g, provided by the University animal house, Babol, Iran. The rats were maintained under a 12 : 12 h light/dark cycle, at temperature: 22 ± 2°C, and humidity: 55 ± 10%. Moreover, rats had free access to food and water. Animals were acclimatized for a period of at least 7 days before the administration. The handling of animals and all experimental procedures were conducted in compliance with the guideline for the care and use of laboratory animals in Iran, and the protocol was approved by the Institutional Review Board of Babol University of Medical Sciences.

#### 2.2.3. Treatment and Blood Sample Preparation

Rats were randomly divided into two groups (*n* = 6): control and pretreatment. The control group received daily injections of physiological saline, whereas the pretreatment group received ketorolac (20 mg/kg/day, intraperitoneal) for 14 days. On the 15th day, blood samples were first collected from the retro-orbital sinus of rats (zero time). Then, rats in both groups received 5-FU (50 mg/kg, intraperitoneal). The blood samples were collected from the retro-orbital sinus of rats at 5, 15, 30, 60, 120, and 240 mins after 5-FU injection. Then, plasma was carefully separated. Plasma, 5-ClU (50 *µ*g/ml as internal standard), and acetonitrile were poured into microtubes, in that order. The contents of the microtubes were made completely uniform by vortex for 1-2 mins and then centrifuged at 4°C and 16000 RPM for 10 mins. Supernatants were collected and transferred to new microtubes, and then the microtubes were refrigerated with the lid open to evaporate the solvent. Finally, methanol was added to the contents of each microtube and the mixture was vortexed for 1-2 mins before injection into the HPLC system.

#### 2.2.4. HPLC Assay of 5-FU in Rat Plasma

High performance liquid chromatography (HPLC) is a simple, fast, and sensitive method that has been developed for plasma 5-FU analysis and has been validated for pharmacokinetic studies [14]. In this study, HPLC device with UV detector (maximum wavelength 264 nm) was used. Separation was performed using C18 column at 25°C. The mobile phase consisted of ammonium acetate buffer (0.01 M, pH = 3.5) and acetonitrile, which was pumped at a flow rate of 0.75 ml/min.

#### 2.2.5. Pharmacokinetic Parameters and Statistical Analysis

Pharmacokinetic parameters for each rat were calculated using PK-Solver software based on a two-compartment model. Comparisons between groups were carried out by *t*-test. The significance level was set at *p*-value ≤0.05.

## 3. Result

### 3.1. Cell culture

**Figure 1:**
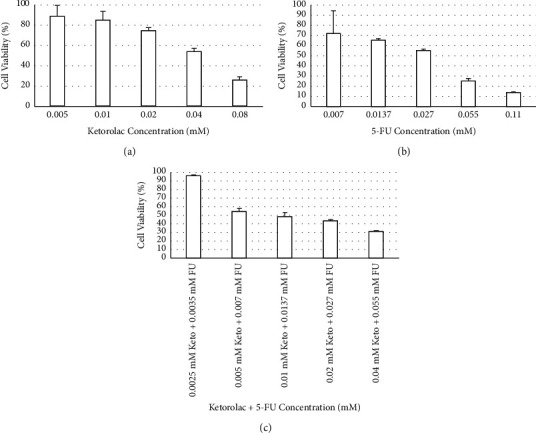
Viability (%) of HT-29 human colorectal cancer cells after 48 hours of treatment with various concentrations of ketorolac (a), 5-FU (b), and combination of them (c). Cell viability was determined by MTT assay and evaluated using a cell viability index (%), which was defined as: (mean absorbance in the test group/mean absorbance in the negative control group) × 100. Results are mean ± SD and significance level set at *p* ≤ 0.05.

The results are reported as mean ± SD (standard deviation). No significant difference was detected after 24 hrs of treatment with ketorolac, 5-FU, and ketorolac +5-FU. The results of 48 hrs of treatment are as follows: the percentage of cell viability at concentrations of 0.005, 0.01, 0.02, 0.04, and 0.08 millimolar (mM) ketorolac were 89.04 ± 10.94, 85.15 ± 8.80, 75.00 ± 3.11, 54.18 ± 3.11, and 25.88 ± 3.26, respectively. The percentage of cell viability at concentrations of 0.007, 0.0137, 0.027, 0.055, and 0.11 mM 5-FU were 72.24 ± 22.73, 65.76 ± 1.65, 55.64 ± 1.17, 25.29 ± 2.53, and 14.33 ± 0.34, respectively. The percentage of cell viability at concentrations of 0.0025 mM ketorolac +0.0035 mM 5-FU, 0.005 mM ketorolac +0.007 mM 5-FU, 0.01 mM ketorolac +0.0137 mM 5-FU, 0.02 mM ketorolac +0.027 mM 5-FU, and 0.04 mM ketorolac +0.055 mM 5-FU were 96.04 ± 0.73, 54.02 ± 3.94, 48.09 ± 5.3, 43.48 ± 1.26, and 31.36 ± 0.84, respectively (Figure 1).

**Figure 2:**
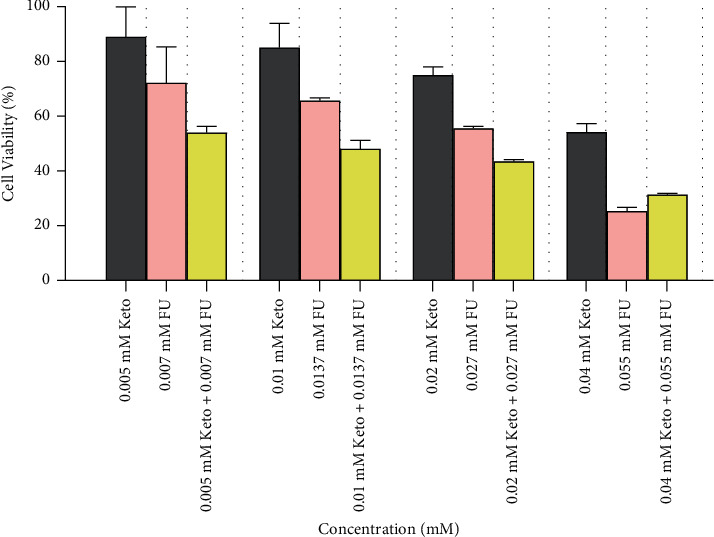
Comparison of HT-29 human colorectal cancer cell viability (%) after 48 hours of treatment with ketorolac, 5-FU, and combination of them at almost similar concentrations. Cell viability was determined by MTT assay and evaluated using a cell viability index (%), which was defined as: (mean absorbance in the test group/mean absorbance in the negative control group) × 100. Results are mean ± SD and significance level set at *p* ≤ 0.05.

According to the results of the MTT assay, the percentage of cell viability decreased in proportion to the increase in drug concentration in each group. The rate of reduction in viability after treatment with 5-FU was higher than ketorolac (at almost similar concentrations). In a certain concentration range, the rate of reduction in viability after combination therapy with ketorolac and 5-FU was higher than ketorolac or 5-FU (at almost similar concentrations), and combination therapy with these drugs showed synergistic effects: 5-FU + ketorolac >5-FU > ketorolac. However, at concentrations above this range, combination therapy showed antagonistic effects on 5-FU function, and the rate of reduction in viability after treatment with 5-FU was higher than combination therapy or treatment with ketorolac (at almost similar concentrations): 5-FU > 5-FU + ketorolac > ketorolac (Figure 2).

### 3.2. Pharmacokinetic Analysis

**Figure 3:**
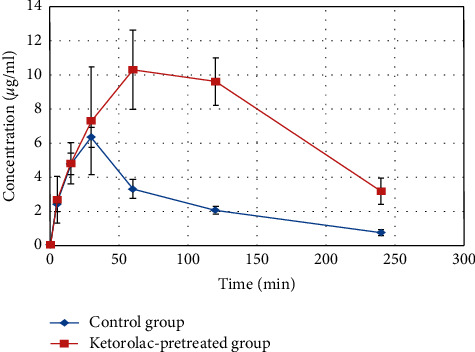
Mean ± SD of plasma concentration of 5-FU (50 mg/kg, i.p.) in the control group and the ketorolac-pretreated group.

The observed plasma concentrations of 5-FU after intraperitoneal injection of 5-FU (50 mg/kg) to the control group and the ketorolac-pretreated group are presented as mean ± SD in Table 1. The mean plasma concentration-time curves of 5-FU (50 mg/kg, intraperitoneal) in the control group and the ketorolac-pretreated group are shown in Figure 3, and the standard deviation is specified on it. The pharmacokinetic parameters calculated based on the two-compartment model and using PK-Solver software are presented as mean ± SD in Table 2. *K*_a_, K_12_, K_21_, and K_10_ show the absorption, distribution, redistribution, and elimination rate constants, respectively. The amount of these constants in the pretreatment group was significantly lower than the control group (*p* < 0.05). *T*_1/2*α*_ and *T*_1/2*β*_ indicate the distribution and the elimination half-life, respectively. Based on the analysis, *T*_1/2*α*_ in the pretreatment group increased significantly (by an average of 366%) and *T*_1/2*β*_ decreased (by an average of 5%). Changes in *T*_1/2 *β*_ were not noticeable. However, considering the drug concentration profile in rats pretreated with ketorolac, it seems that the time allotted for this study (4 hours) was not adequate, and decision on the pharmacokinetic behavior of 5-FU in the elimination phase requires further studies. The volume of distribution over bioavailability (V/F) and apparent clearance (CL/F) of 5-FU in the pretreatment group compared to the control decreased significantly by an average of 32% and 66% (*p* < 0.05), respectively. In this study, it was also found that the maximum plasma concentration (*C*_max_) and time to reach it (*T*_max_) and also the area under the curve (AUC) from time zero to the end of sampling (AUC_0-240_) in the pretreatment group compared to the control increased significantly by an average of 83%, 173%, and 208% (*p* < 0.05), respectively (Table 1).

## 4. Discussion

Based on previous studies, combination of 5-FU with various modulators including DPD inhibitors may enhance the apoptotic and antitumor effects of 5-FU. Studies have shown that the NSAIDs can be promising modulators for 5-FU-based chemotherapy, especially in tumors with high COX-2 expression. COX-2 is the best target characteristic of NSAIDs. The activity of DPD and COX-2 is significantly increased in inflammatory diseases and various malignancies, including colon cancer. The coexistence between COX-2 activity and DPD has been proven, and the effect of NSAIDs on these enzymes has been studied. According to these studies, treatment with NSAIDs at low doses reduces COX-2 and DPD activity simultaneously [15]. Inhibition of DPD gene expression or loss of its enzymatic activity leads to accumulation of 5-FU chemotherapy drug, which leads to a significant increase in drug toxicity. In addition to inhibiting COX-2 and DPD, some NSAIDs can potentiate the inhibitory effects of 5-FU on tumor growth in parallel with the induction of apoptosis by inhibiting thymidylate synthase or increasing the expression of p53, c-jun, caspase-3, Bax, etc. Based on the results of cell culture in the present study, ketorolac is able to reduce the viability of cancer cells, which confirms the role of NSAIDs in cancer management. On the other hand, it was found that combination therapy with ketorolac and 5-FU in a dose-dependent and time-dependent manner has synergistic effects on reducing the viability of cancer cells. However, further studies are needed to understand the mechanism of action of ketorolac in inducing cell death and the mechanisms involved in causing this synergistic effect.

According to various studies, NSAIDs are able to alter the pharmacokinetics of other drugs, including some chemotherapeutic agents. These drugs may significantly affect the disposition kinetics of other drugs. They can also separate other drugs from the plasma protein binding site, inhibit their metabolism, or impair renal excretion [16]. Lack of effectiveness and incidence adverse effects and toxicity are considered to be the main causes of treatment failure. The pharmacokinetics of drugs are largely related to these causes [17]. Although 5-FU is a prodrug and it seems difficult to establish a direct relationship between the pharmacokinetics of this drug and its effectiveness or toxicity, the results of several studies have shown that there is a close relationship between plasma concentration (and pharmacokinetic parameters such as AUC and clearance) of 5-FU and its effectiveness or toxicity [18, 19]. Therefore, pharmacokinetic evaluation will help ensure that treatment will not fail and it will be safe. The results of the present study indicated a pharmacokinetic interaction between ketorolac and 5-FU. In other words, ketorolac is able to alter the pharmacokinetics of 5-FU. According to the abovementioned, it seems that these changes are towards creating a synergistic effect on 5-FU cytotoxicity.

5-FU, like other antimetabolite agents, has the highest activity in the S-phase of the cell cycle [20], and its antitumor effects depend on the duration of drug exposure more than its plasma level [21]. Based on the results of pharmacokinetic analysis, the presence of ketorolac in the 5-FU chemotherapy protocol may have a positive effect on both time and concentration factors. On the other hand, previous studies have shown that an acute increase in 5-FU plasma concentration can cause severe side effects [22]. Therefore, it seems that ketorolac can partially prevent the severe side effects arising from the acute increase in 5-FU plasma concentration. Furthermore, in this study, contrary to what is usually seen in the clinic, intraperitoneal injection method was used to administer 5-FU. Although there is more pharmacokinetic diversity following intraperitoneal administration compared to the intravenous injection, this method may have advantages such as increasing the drug concentration in the peritoneal cavity, high drug concentrations in the portal vein blood, and reducing systemic toxicity. Comparison of intraperitoneal administration with intravenous administration of 5-FU as adjunctive chemotherapy in patients with colorectal cancer has shown that due to the reduction of hepatotoxicity and hematological toxicity in the intraperitoneal route, higher doses of the drug can be administered through this way [23]. Accordingly, pretreatment with ketorolac as well as the choice of intraperitoneal administration method both affect 5-FU-induced toxicity.

The pharmacokinetics of 5-FU are significantly influenced by various factors such as high serum alkaline phosphatase level, drug infusion duration, and DPD level [19, 24]. DPD is an effective enzyme in the first step of reduction and the rate-limiting step in the degradation of pyrimidine nucleic acids and their analogues. Although cytochrome P-450 isoenzymes are the most abundant drug metabolizing enzymes in phase I, other enzymes such as DPD also affect the pharmacokinetics and effectiveness of drugs [25]. DPD is a key enzyme in the 5-FU catabolism process, and more than 80% of the administered dose of 5-FU is broken down by this enzyme in vivo. The activity level of this enzyme is one of the most important determinants of 5-FU toxicity. DPD, like other enzymes involved in drugs metabolism, may be altered as a result of concomitant use of other drugs. Kobuchi showed that the factor that significantly affects the pharmacokinetic parameters of 5-FU (such as AUC, total clearance, and half-life) after administration of single or repeated doses of 5-FU is hepatic DPD activity [26]. Reference [27] showed that nonsteroidal anti-inflammatory drugs in combination with 5-FU by DPD modulation (decreased mRNA expression or enzymatic activity) can simultaneously and significantly increase the sensitivity and cytotoxic effects of 5-FU in xenografts and cells expressing high COX-2(27). Therefore, it seems that one of the possible mechanisms of ketorolac in creating a synergistic effect on the efficiency of 5-FU in reducing the cancer cells viability and causing pharmacokinetic interaction with it is DPD modulation. Other possible mechanisms involved in causing pharmacokinetic changes are the inhibitory effects of ketorolac on renal secretion and the reduction of 5-FU glomerular filtration via inhibition of prostaglandin synthesis [28, 29]. Our study had some limitations. In this study, we evaluate the 5-FU serum concentration, and we did not evaluate its metabolites which are responsible for its anticancer and toxic effects. Moreover, we cannot define the absolute mechanisms for the observed effects of ketorolac. More studies are needed to understand the mechanism of the effectiveness and possible side effects.

These results are important because, first, in a group of patients who do not respond well to 5-FU at therapeutic doses, while using ketorolac as an analgesic in the treatment protocol, in addition to reducing the dose and side effects of opioid analgesics can increase the effectiveness of 5-FU without need for increasing the dose. However, further studies are needed to evaluate the toxicity of this drug regimen. Second, in a group of patients who suffer from 5-FU-induced toxicity at therapeutic doses due to polymorphism in the DPD gene, recognizing the interaction between these drugs will help adjust the individual dose and prevent unwanted side effects.

## Figures and Tables

**Table 1:** Plasma concentration of 5-FU (50 mg/kg, i.p) in the control group and the pretreatment group as mean ± SD (*p* < 0.05).

Time (min)	Mean ± STDEV plasma concentration of 5-FU (*µ*g/ml)	
	Control group (5-FU alone)	Pretreatment group (5-FU + ketorolac)
0	0	0
5	2.39 ± 0.45	2.67 ± 1.38
15	4.74 ± 0.63	4.79 ± 1.21
30	6.31 ± 0.59	7.29 ± 3.16
60	3.28 ± 0.55	10.28 ± 2.31
120	2.04 ± 0.24	9.58 ± 1.40
240	0.72 ± 0.17	3.15 ± 0.77

**Table 2:** Pharmacokinetic parameters of 5-FU (50 mg/kg, i.p) in the control group and the pretreatment group as mean ± SD.

Parameter	Control group (5-FU alone)	Pretreatment group (ketorolac + 5-FU)
Ka (1/min)	0.046 ± 0.002	0.023 ± 0.011
K_10_ (1/min)	0.018 ± 0.001	0.01 ± 0.002
K_12_ (1/min)	0.019 ± 0.001	1.81E-10 ± 1.3E-10
K_21_ (1/min)	0.011 ± 0.001	0.005 ± 0.002
*T *_1/2Alpha_ (min)	15.99 ± 0.67	74.44 ± 14.66
*T *_1/2Beta_ (min)	153 ± 8	145 ± 58
*T *_1/2ka_ (min)	14.95 ± 0.63	37.3 ± 18.1
V/F ((mg/kg)/(*μ*g/ml))	3.74 ± 0.41	2.54 ± 0.89
CL/F ((mg/kg)/(*μ*g/ml)/min)	0.068 ± 0.011	0.023 ± 0.005
*T *_max_ (min)	25.65 ± 0.52	69.95 ± 13.77
*C *_max_ (*μ*g/ml)	5.69 ± 0.68	10.39 ± 1.49
AUC 0–240 (*μ*g/ml.min)	568 ± 78	1750 ± 280
AUC 0-inf (*μ*g/ml.min)	750 ± 115	2206 ± 374
AUMC (*μ*g/ml.min^2^)	130199 ± 24878	356887 ± 70025

## Data Availability

The data supporting the findings of this study are available on request from the corresponding author.
